# A Systematic Treaties, Historical, Etiolgical, and Practical, of the Principal Diseases of the Interior Valley of North America

**Published:** 1855-05

**Authors:** 


ART. V.—*A Systematic Treatise, Historical, Etiological, and Practical, of the Principal Diseases of the Intereor Valley of North America, as they appear in the Caucasian, African, Indian, and Esquimaux varities of its population.* By DANIEL DRAKE, M. D. Edited by S. HANBURY SMITH, M. D., Professor of the Theory and Practice of Medicine in Starling Medical College, Ohio, and FRANCIS G. SMITH, M. D., Professor of the Institutes of Medicine in the Medical Department of Pennsylvania College, Philadelphia. Second Series. Philadelphia. Lippincott, Grambo & Co. 1854.

---

The gifted author of this work, as is known to all our readers, was cut off in the midst of his labors. His first volume had been published and he had advanced far in the completion of the second when he was called to resign his task into the hands of others. The editors of his papers have executed the trust committed to them faithfully and industriously. We regret that a more popular house was not chosen for its publication, but this ought not to prejudice the sale of a work intrinsically so valuable.

This treatise was the great labor of Dr. DRAKE's industrious life. He had meditated a work on the diseases of the great central valley of America for more than thirty years. Ten years before he died he had collected a great mass of materials for such a work, and for a number of years he traveled extensively to acquire a personal knowledge of the medical topography of the country and of the diseases which he was about to describe. His success was commensurate with his immense toil. On all hands, his work, when it appeared, was pronounced the most creditable to our medical literature that any American pen had produced.

The second series will be hailed with uncommon pleasure by a large class of readers. It goes far towards completing the plan of its lamented author, and forms a volume which ought to find a place in the library of every American physician. The first volume consisted mainly of an account of the topography and climate of the country, concluding with a very thorough description of autumnal fevers. The editors of the second volume have wisely concluded to republish these concluding chapters, giving in one volume all that Dr. DRAKE had written on the subject of fevers.

An elaborate discussion of yellow fever occupies nearly two hundred of these closely printed pages, which will be consulted by every reader who desires full information on the subject. Dr. DRAKE himself never saw a case of yellow fever, yet, as he says, he was "not precisely in the condition of a historian, who might have only printed works from which to compile," for he visited and studied "the topography, hydrography, and climate of a number of the most infested localities," and, what he regarded as of equal or greater advantage, saw and conversed with a number of physicians, who had become familiar with the disease in all its aspects. Under the head of geography and chronology of the disease, he gives the number of times that it has prevailed sporadically or as an epidemic in the Valley of the Mississippi river and its borders, and from his table it appears that up to 1849 no year had passed since 1817 without more or less of the fever. The history of the fever in this region reaches back as far as 1702. We cannot follow the author into details respecting the local history of the disease, which however possess great interest and will reward the careful study of the etiologist. After presenting a long array of facts on this subject, he makes his etiological deductions which we copy in extenso.

"Let us now proceed to ascertain the bearings of the facts which have been set forth, on the origin and spread of yellow fever.

"Passing by the vexed and difficult inquiry, whether the yellow fever of Havana originated there, or was introduced from abroad, we may speak and treat of it as a disease of that city; seeing that although it is not extensively prevalent every summer, it is never absent, and thus, if not a native, is a naturalized endemic. In reference to the first case that ever occurred in that city, it may be said that if the cause was generated there, the subsequent prevalence of the disease has no doubt depended on the continual generation of the same cause.

"If the disease was imported, we may assume, with equal confidence, that the introduction and continuance of the fever have depended on one of two causes,—either a contagions emanation from the bodies of the sick, in perpetual succession, or a peculiar fermentative power in the portion of empoisoned air originally imported, whereby it transformed that of Havana into its like, and has ever since kept up the disease.

"But the same views are not applicable to New Orleans, where a colder winter might destroy the cause of an imported disease, which would not re-appear till it was re-introduced. Hence the fever may prevail annually in Havana, although originally an exotic, but be frequently extinguished in New Orleans, and require to be imported anew.

"Our first inquiry relates to contagion. In the prosecution of this, the rules of logic require the exclusion of all cases in which the disease is said to have been contracted by going on board of a ship or boat where there were yellow fever patients, or by visiting a patient in a town affected with the fever when it is epidemic, for the individual in these cases is exposed to the same atmosphere which may have produced the fever in the sick, and it cannot be told whether he contracted the fever from them, or from entering the same localities which had occasioned it *in* them. We must also, for the same reason, exclude the cited instances of epidemic fever soon after the arrival of a ship or boat, because it may have brought a portion of the atmosphere from the sickly port whence it sailed, or may have arrived at the moment when the disease was about to appear from local causes. Thus restricted, the number of facts, going to show the propagation of yellow fever by contagion is surprisingly small, and most of them far from being unquestionable.

"1. When speaking of the introduction of the yellow fever into Opelousas, in 1837 and 1842, we gave an account from Prof. Carpenter's book of the apparent successive propagation of the disease from two strangers who arrived there from New Orleans and sickened of the fever. It will be recollected that steamboats do not reach this town. But it must be remembered that in the former of these years the disease was extensively prevalent; in the latter, however, it was limited to New Orleans and Mobile.

"2. When the body of Dr. Smith, of Plaquemines, was taken to New Iberia, it was said there was no fever in the village, but that nearly all who visited the church in which the corpse was deposited or attended the funeral, were soon afterwards seized with the fever. This was in the year 1839, when the disease was more extensively prevalent than in any other year.

"3. Under the head of St. Francisville there are details which show an apparent propagation of the fever from one person to another; but the fever that year was extensively epidemic, and previously to the occurrence of the second link in that chain of cases, it was prevailing in the adjoining village of Bayou Sara.

"4. When treating of the epidemic of Woodville, we found that on a certain plantation, a number of negroes who had not visited the town experienced attacks of the fever after the death of one who had contracted it there; but the author of this important observation likewise informs us, that the disease occurred on some plantations, where, as far as he could ascertain, it had not been introduced. There were, indeed, signs of an epidemic constitution for several miles around Woodville.

"5. By referring to the history of the yellow fever in New Orleans, for the year 1841, the reader will find an account from Dr. Beugnot and Luzenburg, of its apparent introduction and spread by officers and seamen of the Talma, anchored off La Fayette, adjoining the city, where, as they state, the fever had not in any previous season commenced.

"If the history of the disease for the last fifty years in the Valley of the Mississippi furnishes any other facts going to show the contagiousness of yellow fever, I have not been able to find them. There are, however, two analogical arguments in support of this opinion, which must not be overlooked.

"1. The fever is not intermittent, but approaches to the continued type. Now typhus and typhoid, which are continued fevers, are known (or believed) to generate contagion; and from analogy we might predicate the same of yellow fever.

"2. We have seen as a general fact that yellow fever affects persons but once, which, as all the world knows, is true of the eruptive fevers, and as they are contagious, we might conclude that yellow fever is the same. But on this method of analogical reasoning, yellow fever ought to be eruptive, as well as contagious.

If it fail in one it may fail in the other also. The relation between eruption and contagion is in fact much closer than between immunity from second attacks and contagion, for typhus fever and syphilis are contagious and eruptive, yet second attacks of the former are not uncommon, and it is not proved that an attack of the latter bestows any immunity against future infection.

"There is, moreover, a difference between yellow fever and the true eruptive fevers, as to exemption from second attacks; for residence in a cold climate destroys it in yellow fever, but no change of climate has that effect in the eruptive fevers. Thus the contagiousness of yellow fever cannot be established by this any more than by the other.

"Let us now examine the facts which oppose the doctrine of contagion.

"If we look to small-pox, measles, and scarlatina, the best examples of contagious fevers, or even add to them typhoid and typhus fevers, we find that they propagate themselves in all seasons, localities, and conditions, provided the emanations from the body of the patient be not decomposed or wafted away; but such is not the case with yellow fever.

"1. Thus, vessels arrive in New Orleans in the months of April, may, and June, from the Havana, when yellow fever is prevailing there, but the cases of fever which they bring, do not propagate the disease. Yet if contagion exists, why is it not as operative in those months as in August and September? It certainly differs from all other contagions, if it cannot exert itself as well *before* as *after* the summer solstice.

"2. As soon in autumn as the cold becomes great enough to freeze the ground, yellow fever ceases, but cold does not, any more than heat, put a stop to small-pox, measles, and typhoid fever. And here again yellow fever fails to stand the test.

'3. The fevers just mentioned prevail both in town and country; but yellow fever is almost limited to the former;—another failure.

"4. Small-pox, measles, and scarlatina, are propagated and become epidemic in salubrious, as readily and certainly as insalubrious localities; which is not true of yellow fever.

"Thus we see that this disease does not conform to the laws of contagious fever. But stronger facts remain to be stated.

"Nothing connected with the whole subject is more definitively settled than that persons may die of yellow fever, both in town and country, without communicating the disease. Many accurately-observed and well-attested cases of this kind are scattered through our historical narratives; and a much greater number might have been given. Now, such events in such places are the true tests of the contagiousness of this disease, for no other alleged cause is present. In New Orleans and other commercial cities, in the latter part of summer and early autumn, there is another cause; and how is it possible to analyze the phenomena, and say that one depends on that cause, and another on contagion? It may be said, however, that the city atmosphere is not the direct cause of any of the phenomena, but the cause of the contagiousness of the fever. Thus, if two individuals in the same ship had received the *semina* of the fever in Havana, in the month of August, and one should be landed on a plantation below the city, and the other brought into it, and both should sicken, the city atmosphere would so act on the system of one as to cause it to secrete contagion, while the system of the other, surrounded by a pure air, would not secrete contagion—one would communicate the disease, the other not. As I know of no fact or analogy going to support this hypothesis, I am compelled to reject it; but must not do so without remarking that it recognizes the necessary, though indirect, agency of a particular local condition of the atmosphere in the production of the fever.

"But another view may be taken of this matter, which is, that the *predisposing* impress of impure air is a necessary condition to the action of the contagion, and consequently, that it produces no effect beyond the limits of such an atmosphere, and therefore, *seems* not to exist, when it really does. According to the preceding view, contagion is not produced without the influence, *on the body of the sick*, of an impure atmosphere. According to this view, contagion is inert with the predisposing influence of an impure atmosphere *on the bodies of the healthy*. This recognizes the action of two causes, and if either be absent, the fever will not appear. It may not be called the hypothesis of compromise, for it tolerates, or rather requires, all that both parties demand. As we have many examples of co-operative and successive influence of remote causes in the production of disease, this hypothesis wears a most plausible aspect, and has drawn to itself nearly all who once held to the doctrine of the sufficiency of contagion, and not a few of those who formerly believed in local causes exclusively. It accounts, in a general way, for the absence of the fever from filthy, but insulated towns, where but one of the causes exists; for its frequent prevalence in commencial towns, which hold intercourse with those further south; for its not appearing in towns which are free from impurities, although cases of yellow fever may be introduced into them; for its prevalence in autumn, when the air from the heat of summer is most impure, and for its cessation in winter, when the further development of exhalations is arrested by frost. It inculcates cleanliness to prevent the development of the predisposing cauee, and non-intercourse with places where the fever is prevailing, to exclude the exciting cause; prudently attending to both, lest the efforts to suppress either should be unsuccessful.

"Now the objections to this hypothesis are:—

"1. That it combines an admitted and an assumed cause; the local contomination is a fact—the contagion an assumption.

"2. That in many invasions of the fever cases have occurred under circumstances which almost preclude the possibility of communication with the sick, and must therefore be referred to the admitted local cause.

"3. That the fever often appears almost simultaneously in various parts of a town, in the same manner as epidemic cholera, or as autumnal fever among the inhabitants of the country.

"4. That it has (as we shall hereafter see) commenced and prevailed in ships, and in some localities on land where the alleged exciting cause, contagion, could not possibly be.

"5. That having admitted one cause, which appears sometimes to have been sufficient, of itself, for the production of the disease, it is unphilosophical to admit another, the existense of which has not been proved.

"There is however anotner view which avoids the last objection, while it comprehends both local contamination and contagion. According to this hypothesis, contagion acts upon the impurities suspended in the atmosphere of places where and when the fever prevails, and excites in them an intestine or fermentative action, which transforms them into its own nature. This hypothesis of contagious fermentation proceeds on three assumptions: first, that contagion is a ferment; second, that the gas or exhalation, which contaminates the atmosphere, is susceptible of fermentation; third, that the produch is the same as the ferment which excited it. If either of the former of these assumptions fail, the hypothesis likewise fails, for there will be no fermentation. But not so with the latter; for it may be that the exhalations from the bodies of yellow fever patients never produced the disease in others by their direct action, are not in fact contagious, but only ferments, capable of transforming the impurities of the atmosphere into the true and only cause of the fever.

"The hypothesis of fermentation certainly avoids some of the objections which lie against that of contagious propagation by personal intercourse, as we may admit, that it can go on rapidly and in a short time transform the ærial impurities of a city into a poison, like itself, so that the fever may be produced simultaneously in various parts of the locality; it also explains the production of a fever atmosphere in a particular portion of a city; and finally accounts for the production of the disease in those who only ride through that part, which the doctrine of personal contagion does not.

"Nevertheless, it must not be forgotten that this hypothesis rests entirely on assumptions, as indeed the whole doctrine of gaseous or ærial fermentaiion does, and is, moreover, liable to some objections which I shall proceed to state. But previous to doing this, it will be proper to bring forward and incorporate with this, another branch of our subject. In the beginning of our inquiry, it was stated hypothetically, that if yellow fever were originally introduced into Havana, and thence upon this continent, it might have been done either by the importation of contagion, or of a portion of that atmosphere, or rather of its like, which produced the first case, and which acted as a ferment in the air of Havana. Now what I am about to say, will apply equally to both these alleged ferments, and embrace every possible case of importation. When a ship arrives in New Orleans from a yellow fever port, and brings patients with that disease, she has, we may admit, within herself, all the causes which prevail in the port from whence she sailed, the local summer atmosphere and the emanations which the sick send forth; and those who visit her may contract the disease precisely as if they had visited the town from whence she sailed; but to make her arrival the cause of a subsequent epidemic, it is assumed that her foul atmosphere is the immediate cause of a transformation of the impurities suspended in the atmosphere of the city into the same kind of poison with itself, and thus she raises an epidemic. She does not merely add her own poisonous air to the impurities already existing in the locality, but impregnates them with a fatal leaven.

"Now can these alleged ferments act on every kind of gaseous impurity? Certainly not. For when introduced into the atmosphere which produces autumnal fever, they excite no fermentation, effect no transformation, occasion no yellow fever beyond their own limits. When yellow fever is epidemic in Mobile, for instance, steamboats run daily up the Alabama river, and lie at the wharves of Kahawba, Selma and Montgomery, while their inhabitants are suffering under remittent and intermittent fever, yet no yellow fever appears. At New Orleans, while yellow fever is raging in the city, and especially near the river, the environs which lie contiguous to the cypress swamp in its rear, are ravaged by remittent and intermittent fevers. Lastly, up the Mississippi river, it is chiefly in the older towns, where the malarial impregnation has become feeble, and autumnal fevers are comparatively rare, that yellow fever makes its appearance. If this were not the fact, the leaven of yellow fever would transform the malaria of autumn into the cause of that disease, and spread it over the whole country. We may take it then as a settled fact that the yellow fever leaven exerts its transforming power only on the impure air of certain localities, and that in the absence of such impurities, there is and can be no spread of that disease. We have then in this state of the atmosphere over yellow fever localities a *conditio sine quâ non* to the spread of that disease from the vessels which import the alleged ferments. But is this malaria of our commercial towns and cities inert and harmless of itself? By no means. For when and where yellow fever does *not* occur, these localities in summer and autumn are infested with a remittent fever, which bears so striking a resemblance to the remittent fever of the country, that every physician regards them as the offspring of the same cause in a state of modification. In the country this agent is generated in swamps, and other humid places, where the recrements of plants and insects are subjected to decomposition, in the towns where yellow fever oftenest prevails, other vegetable recrements, animal exuvia, and exhalations and secretions from a dense population, are subjected to the same decomposition. As the decomposable materials are not identical, the products of fermentation cannot be identical. The rural malaria produces one modification of fever, the civic another. Now is it not remarkable, when these malaria approach so near to perfect identity, that the imported ferment should readily change the civic malaria into yellow fever gas, but produce no effect whatever on the rural malaria? When the two, judging by their effects, so far from being distinct species are not even striking varieties, how is it that the foul air of Havana or the exhalations from the body of a yellow fever patient can start one into active and deleterious fermentation and be utterly inert when mingled with the other? The hypothesis of ferments is bound to answer this question; or else to establish itself by positive facts.

"This latter it has attempted to do in the following manner:—

"1. Ships from Vera Cruz or the Havana have almost always arrived in New Orleans just before an outbreak of the fever.

"2. During the war with England, when the intercourse with the more southern ports of the Gulf was suspended, or nearly so, the fever was absent.

"3. Those places of the Gulf which have most intercourse with Havana, are oftenest invaded

"4. The disease has almost confined itself in the interior to the towns which are visited by steamboats, and has occurred oftenest in those which are oftenest visited.

"5. They have frequently been invaded immediately after the arrival of a boat from New Orleans.

"6. The towns of the interior are not in general affected till the disease has prevailed for some time in New Orleans.

"7. It never appears in any of the towns of the interior when it is absent from New Orleans.

"8. It generally begins near the wharves.

None of these facts can be questioned, but it is proper to estimate rigidly their value in reference to the theory which they seem to support, and this I shall proceed to do:—

"1. Ships are at all times arriving at New Orleans from Havana, Vera Cruz, and other southern ports of the Gulf; and, therefore, if the fever depended on a local or indigenous cause, developed in August and September, it could scarcely happen that the arrival of a ship would not be coincident with it.

"2. As to the absence of the disease before and during the war with England, it does not appear that it was absolute, for Dr. Iluestis, in his account of the diseases of Louisiana, and Judge Chamberlain, now of Mobile, but then of New Orleans, assure us that the fever occurred in 1812, after the declaration of war. But its complete absence during that period would not be conclusive, for in 1815 and 1815, the two years which succeeded the three years of war, it was absent; and in many towns, as for example, Mobile, notwithstanding an unrestricted intercourse with both Havana and New Orleans, it has been absent for periods of two, three, and even ten years; Natchez, from 1829 to 1837; Baton Rouge, in which it did not occur from 1829 to 1839, and again was absent to 1843; to which it may be added, that many towns in constant communication with New Orleans, were not visited till 1839; thus effectually destroying the conclusion drawn from the relative absence of the fever during the war.

"3. If it occur oftenest in the towns which have most intercourse with Havana; oftener, for instance, in New Orleans than Mobile, and in the latter, than in Pensacola, the introduction and agency of a ferment is not proved; for in proportion as towns have more shipping, they have more nuisances, and a more crowded wharf population, and therefore send up more of those exhalations on which the ferment is said to act.

"4. If the disease is almost confined to river towns, it may be because those exhalations which are to be fermented are there most copious.

"5. If the disease often shows itself soon after the arrival of a boat from New Orleans, it may be simply because a local influence is then matured. The previous arrivals without the supervention of the fever, show, according to the theory of ferments, that the local atmosphers was not yet impure enough for the alleged fermentation; when its impurity has become great enough for the fermentation, that process may or may not be necessary to an outbreak of the fever.

"6. If the towns of the interior are not in general affected till some time after the disease has prevailed, that may arise from a slower development of the required local atmosphere, and not from a necessary lapse of time for the production of the ferment in the atmosphere of the city. The conditions, including heat necessary to a local contamination, are less active in the smaller towns north of New Orleans than in that city.

"7. That none of the interior towns have it when New Orleans is exempt. This only proves that the causes which exempt that city, to wit., the absence, not of an imported ferment, but a local impurity to be fermented, have been equally operative in the towns of the interior.

"8. If it generally appears in the neighborhood of wharves, it may be becguse the local contaminated atmosphere is there most concentrated.

"It appears then, that the facts admit of a different interpretation, and do not, therefore, establish the existence and importation of a ferment. But to take away the tendered proofs of a theory does not *disprove*, but merely leave it unsupported. In that condition I conceive the doctrine of ferments now to stand; and in the absence of the support by facts, let us inquire into its probability and necessity.

"1. Although I have called it a theory, we see that it is only an hypothesis, an assertion to be proved—an assumption to explain certain facts, which admit of a different interpretation; which, however, it must be granted, may not be the correct one. If, however, they can be differently explained, they can no longer be exclusively applied to the hypothesis to which they were referred, which before it can reclaim them must establish itself by other evidences.

"2. This hypothesis is not sustained by any known analogy, and, therefore, can derive no support from reasonings carried from the known to the unknown.

"3. It multiplies causes without having the necessity for them shown. If a peculiar impure state of the atmosphere is necessary to the action of the alleged ferment, it may be said that the disease arises from that atmosphere itself, independently of any transformation by fermentative action. This is only elevating a condition, which the other hypothesis admits, to the rank of a *sine qua non* in the production of the fever, the cause, and the *only* cause. It is substituting the impure exhalations themselves, before the alleged fermentation, for the products of that fermentation. Their existence is granted, indeed, demanded by the hypothesis of fermentations, for without them the ferments could not spread the disease; but the very existence of the ferment is an hypothesis. If *they* necessarily exist, whenever the fever occurs, they may be expected to produce some effect, for by the very terms of the case, they are impurities, and that effect may be—*the fever.*

"This brings us to the inquiry whether the theory of an indigenous atmospheric contamination can explain all the phenomena connected with the origin and epidemic prevalence of the disease, in doing which I shall take autumnal fever, a non-contagious endemic, for our model.

"1. Whatever may be the mode in which, or the medium by which yellow fever now spreads through a community, or appears successively in different towns, the first case of the disease cannot have been produced by morbid exhalation from the body of one laboring under that disease, and must therefore be regarded as the product of a cause resulting from chemical action on materials composing the earth, or resting on its surface. But if one case could be originated in that way, others may, indeed must, whenever the same conditions exist. The same conditions may not, however, exist but at the spot where the first case was generated, and the disease may everywhere else depend on derivation from those who labor under it. But we have shown as a matter of fact that it is not thus propagated. We have also shown that many facts and arguments stand opposed to the hypothesis of ferments.

"2. If we can show that the disease has arisen independently both of contagion and of ferments, under circumstances which deteriorate the atmosphere with the material on which the ferment is said to act, we have made out a case identical with that which originally produced the fever, and proved that it is still, as at its primitive appearance, a disease of local origin, the offssring of certain conditions, and a necessary occurrence whenever and wherever those conditions exist.

"What, then, are the proofs of this origin?

"1. When treating of the origin of the fever at Pensacola, I have given the history of the generation of the fever on board of three national ships, the Natchez, Vincennes, and Hornet, altogether apart from any external influence; and when giving its history at Donaldsonville, I stated an instance of its being generated in a small boat on a voyage from Baton Rouge to that town, the disease not existing in either. I might greatly extend this catalogue if I choose to introduce cases of a less rigid character, for the number of instances of alleged spontaneous origin on ships, in the Gulf of Mexico, is very great, but I do not wish to employ apocryphal data.

"2. Passing by New Orleans, where the local and commercial conditions are too much mixed for a satisfactory estimate of the influence of either, I may refer to Mobile, as having several times been invaded, in such parts of the city, and under such circumstances, as to convince the whole of its medical men, and all its observing population, that the fever did, indeed, arise independently of importation. I may also refer to Natchez, some of whose epidemics began under circumstances which seem utterly adverse to the hypothesis of importation; likewise, to the country around Washington, where we have the authority of Dr. Branch and Dr. Monette, the latter a powerful advocate of importation, that the disease has occurred from local causes; to Apalachicola Bay, where, according to Dr. Hart, several cases occurred when no ships had arrived from a distance; to Fort Smith, where the disease appears to have prevailed in 1823; to Memphis, where it was epidemic in 1828, and as far as observation could guide those who witnessed it, arose from conditions existing on the spot; finally, in our examination of the origin of the fever in all the times and places where it has prevailed in the Northern Basin of the Gulf, we found that in almost every case which admitted of a strict examination, the asserted importation of the disease could not be made out, but if it were not imported, it was of local origin.

"3. It does seem to me, then, that the fact of a local origin of some cases is as well established as medical facts generally are, and that we are at liberty to reason from it as a fixed *datum.* But does this prove that the disease is indigenous? The answer must be in the negative. For it is *possible* that in all these cases a portion of the ferment may have been introduced long before, and laid dormant for want of that element in the atmosphere on which it is said to act, and it is also possible that when such an atmosphere was generated the ferment took effect. We cannot disprove this, and therefore admitting all the facts which have just been cited, they do not demonstrate that the fever arose from causes entirely local. But although that grade of proof is not attainable, we may certainly approximate to it; for in the first place the very existence of such a ferment is, as we have shown, an assumption; in the second place, as it is represented to have been a product of the South, and as cold suddenly arrests the fever, it seems highly improbable that it could survive the action of several winters; and in the third place, it is contrary to all analogy that it should neither undergo spontaneous decomposition through so long a period, nor evaporate, nor be absorbed by the bodies to which it adhered, through a series of years, and thus be lost. If these reasons do not annihilate the claims of the doctrine of a ferment, it must, I think, be admitted that it reduces them to the very lowest degree of probability.

"4. The occurrence of the fever from local conditions, independent of any foreign agency, being established in even a single instance, the controversy is, *de jure*, at the end, for if it can be thus generated in one case, it may in all, and to say that it is not, is to superadd a hypothetical to a known cause, in violation of the rules of philosophizing. He, indeed, who admits its production in this manner in one instance, and denies it in others, is bound to support his denial,—he brings upon himself the *onus probandi.* When a phenomenon has been once proved to arise from a certain cause, true logic requires us to refer it whenever and wherever it may afterwards appear to the same cause. If then yellow fever on board the ship Natchez, or in the vicinity of Washington, or in the village of Memphis, arose from local causes only, it has arisen in every other town through which we have travelled, from like causes, in other words is indigenous.

"Having made this generalization, let us proceed to inquire whether in its history it conforms to the laws of the endemic fevers with which we proposed to compare it.

"1. It prevails in a certain season of the year, setting in about the time the surface of the earth has attained its maximum heat, and ceasing when the temperature is reduced below a certain point.

"2. While like autumnal fevers it extends indefinitely to the south, it has, like them, shown itself limited to the north, but the limits of the two diseases are not the same, the boundaries of the former being much higher than those of the latter.

"3. Within these limits it invades certain localities and is never seen in others, and the same is true of autumnal fever. From some it is scarcely absent any year, while others are invaded only now and then, and the same is true of them.

"4. In some seasons, as those of 1819, 1827, 1829, and 1843, but above all in that of 1839, it appeared as a widespread epidemic, while in others it has been limited to a few places, or even to New Orleans; and autumnal fever varies in a similar manner.

"5. It is often seen to be influenced in its aspect by the cause of autumnal fever. The cause of typhoid fever has now and then produced modifications in its symptoms. In the year 1811, at Terre aux Bœufs and New Orleans, in the army, it was modified by scorbutus. During the prevalence of epidemic cholera, in the latter, it assumed many of the characteristics of that epidemic.

"The whole of which is true of autumnal fever.

"6. In successive years, at the same place, it presents diversities which cannot be explained; and in the same season, in different localities, variations of character have been noted; which is also the case with autumnal fever.

"Thus conforming to nearly all the laws of our non contagious, endemical, epidemic fever, it presents additional claims to be regarded as such. It meets in fact all the requirements of the theory of indigenous origin.

Is yellow fever, then, it may be asked, merely the remittent autumnal fever with which we are all familiar? The answer according to these views of its origin is, that it is one of the varieties, as a tertian fever is another. That it springs from a peculiar modification of the remote cause in the localities where it prevails; but that this modification is produced by local conditions, not by an imported ferment. That this variation in the character of the agent which produces it, is progressive. That the fever in June and July may wear the aspect and possess the intrinsic pathological nature of remittent autumnal fever, but in August, September, and October, display the more malignant character of yellow fever, returning in November to the milder form of an ordinary remittent.

"Such is the malarial theory of this formidable disease; that which is held by a majority of the physicians of the South;—a theory which inculcates cleanliness and free ventilation in houses, yards, alleys, streets, boats, ships, and wharves, to obviate the development of civic malaria, which being prevented, the fever, according to this theory or according to the theory of an importation of ferments to act on that malaria, would equally fail to appear.

"But I cannot regard this theory as finally established, although obnoxious to fewer objections than the theory of ferments, for,—

"1. The whole pathological history of yellow fever *seems* to constitute it a specific fever, differing from an autumnal remittent too far to admit of its being a mere variety of that disease.

"2. It has occurred in localities where civic malaria can scarcely be admitted to exist, as in Washington and its vicinity, in the state of Mississippi; in Opelousas, and in the Navy Yard on Pensacola Bay, where a white sand drift constitutes the surface of the ground.

"3. It has been produced by exposure to the air, which has escaped from goods sent from a city where the dis ase prevailed. But I know of no facts which go to show that autumnal fever is ever produced in that manner. It is scarcely necessary here to repeat that there is no well-authenticated example of its spreading from this source.

"4. While the condition of a town to all visible appearance continues the same, the disease will appear and disappear, precisely as it does while the commercial intercourse with a place continues unchanged.

"Until these objections are removed, the cause of this disease should be kept *sub judice.* We are not compelled to choose among conflicting theories and declare that which is best supported to be established. It may be more probable than any other, and yet not be true."

